# Supplementary material for: Production of Modified Nucleosides in a Continuous Enzyme Membrane Reactor
Source: Int J Mol Sci. 2023 Mar 23;24(7):6081. doi: 10.3390/ijms24076081 (PMC10094030; doi:10.3390/ijms24076081)
Supplement: Supplementary file 1 [file ijms-24-06081-s001.zip › ijms-2283174-supplementary.pdf]

Supplementary Material

Production of Modified Nucleosides in a Continuous Enzyme Membrane Reactor

Isabel Thiele[1], Heba Yehia[1,2], Niels Krausch[1], Mario Birkholz[1,3], M. Nicolas Cruz Bournazou[1], Azis Boing Sitanggang[4], Matthias Kraume[5], Peter Neubauer[1], Anke Kurreck[1,6,*]

[1] Chair of Bioprocess Engineering, Department of Biotechnology, Technische Universität Berlin, Ackerstr. 71-76, ACK24, D-13355 Berlin, Germany
[2] Current address: Department of Chemistry of Natural and Microbial Products, Pharmaceutical and Drug Industries Research Institute, National Research Centre, Dokki, 12622, Cairo, Egypt
[3] IHP - Leibniz-Institut für innovative Mikroelektronik, Im Technologiepark 25, 15236 Frankfurt (Oder), Germany
[4] Department of Food Science and Technology, IPB University, Kampus IPB Darmaga, Bogor 16680, Indonesia
[5] Chemical and Process Engineering, Technische Universität Berlin, Straße des 17. Juni 135, 10623 Berlin, Germany
[6] BioNukleo GmbH, Ackerstr. 76, 13355 Berlin, Germany

Table of content

Figure S1. Batch reactions for the synthesis of dAdo (A), 6C2FP-dR (B) and 2,6DCP-dR (C) in 5 mL scale or in EMR scale (92 ml). Nucleosides were enzymatically produced at 40 °C, 200 rpm, 25 mM of Thd and 10 mM of sugar acceptor using purified NPs PyNP-02 and PNP-02. 2

Figure S2. Analysis of the reactor content by SDS-PAGE (at t(end)), supernatant and precipitate after centrifugation were analysed (16,000 x g, 4°C, 10 min), marker: p7704 (NEB). Enzyme amounts in the reaction: 150 U PyNP-02; 1050 U PNP-02. 3

Figure S3. HPLC analysis of the EMR permeate. Conversion/concentrations of dAdo and the side products (dIno – 2'-deoxyinosine; Hx – hypoxanthine) were determined. Membrane changes are indicated by asterisks. 4

Figure S4. Transglycosylation reaction to produce the nucleoside analogues 6C2FP-dR (A/B) and 2,6DCP-dR (C/D) using purified NPs PyNP-02 and PNP-02. 25 mM of Thd and 10 mM of sugar acceptor were used as substrates and a reaction temperature of 40°C was applied. Sugar donor cleavage and nucleoside formation were observed in the EMR using a stirring speed of 200 rpm and a residence of 4 h (A/C) or 8 h (B/D) (and 16 h, D). 5

Figure S5. Calculation of the conversion percentage using example chromatograms for dAdo (A), 6C2FP-dR (B) and 2,6DCP-dR synthesis (C). The peak areas were converted to concentrations (dil. concentration) using conversion factors. By calculating the dilution factor (from the preparation of samples for HPLC) out, the concentration in the reaction was determined. The conversion [%] was then determined using equation 6. 6

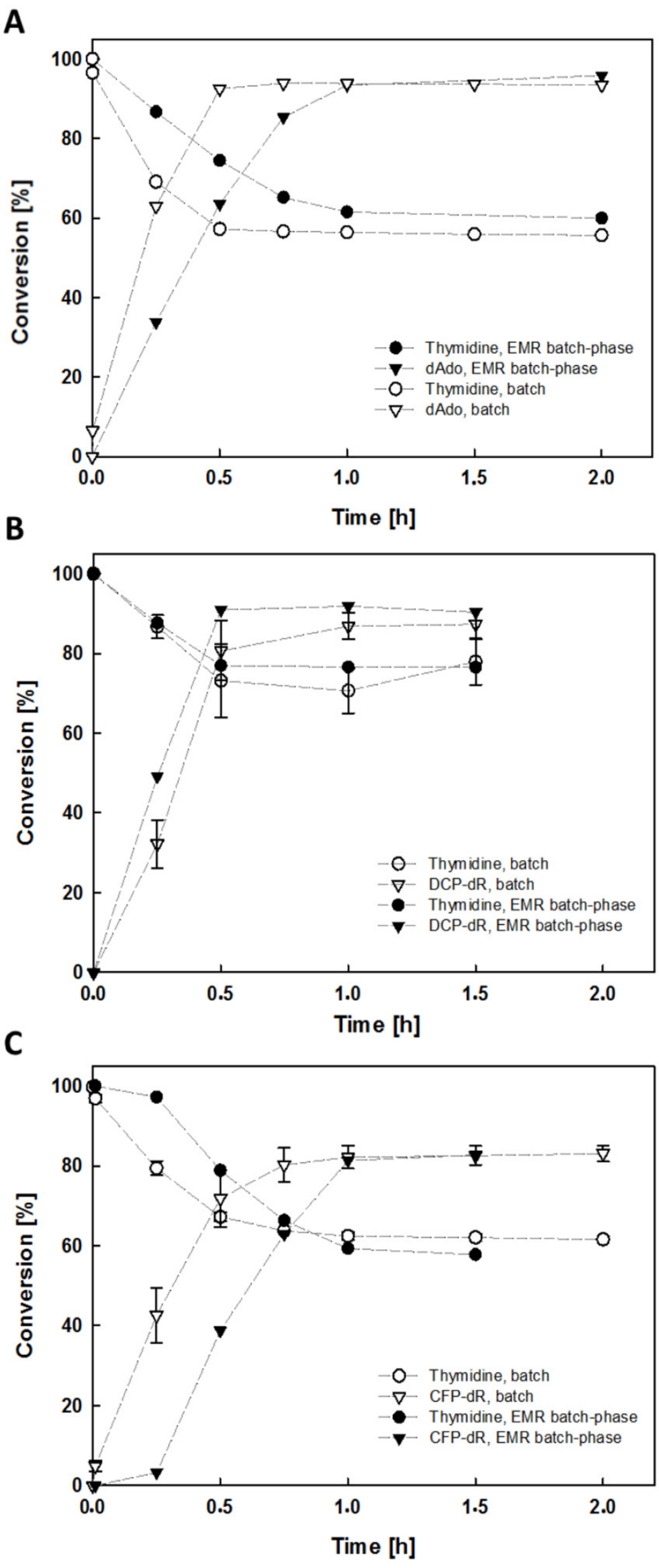

Figure S1. Batch reactions for the synthesis of dAdo **(A)**, 6C2FP-dR **(B)** and 2,6DCP-dR **(C)** in 5 ml scale or in EMR scale (92 ml). Nucleosides were enzymatically produced at 40 °C, 200 rpm, 25 mM of Thd and 10 mM of sugar acceptor using purified NPs PyNP-02 and PNP-02.

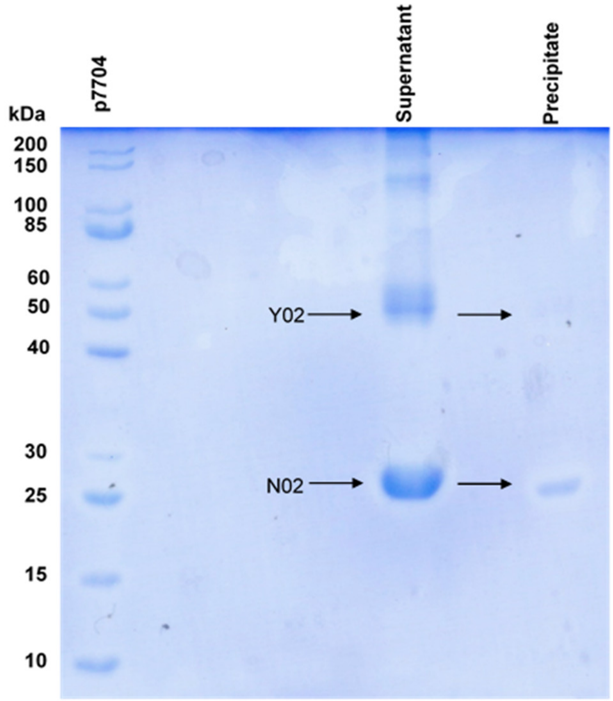

Figure S2. Analysis of the reactor content by SDS-PAGE (at t(end)), supernatant and precipitate after centrifugation were analysed (16,000 x g, 4°C, 10 min), marker: p7704 (NEB). Enzyme amounts in the reaction: 150 U PyNP-02; 1050 U PNP-02.

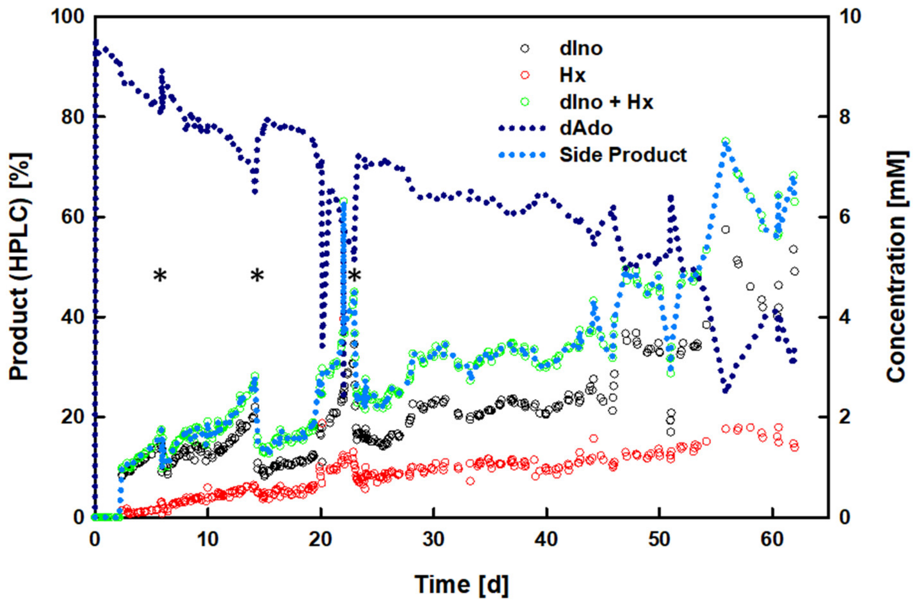

Figure S3. HPLC analysis of the EMR permeate. Conversion/concentrations of dAdo and the side products (dIno – 2′-deoxyinosine; Hx – hypoxanthine) were determined. Membrane changes are indicated by asterisks.

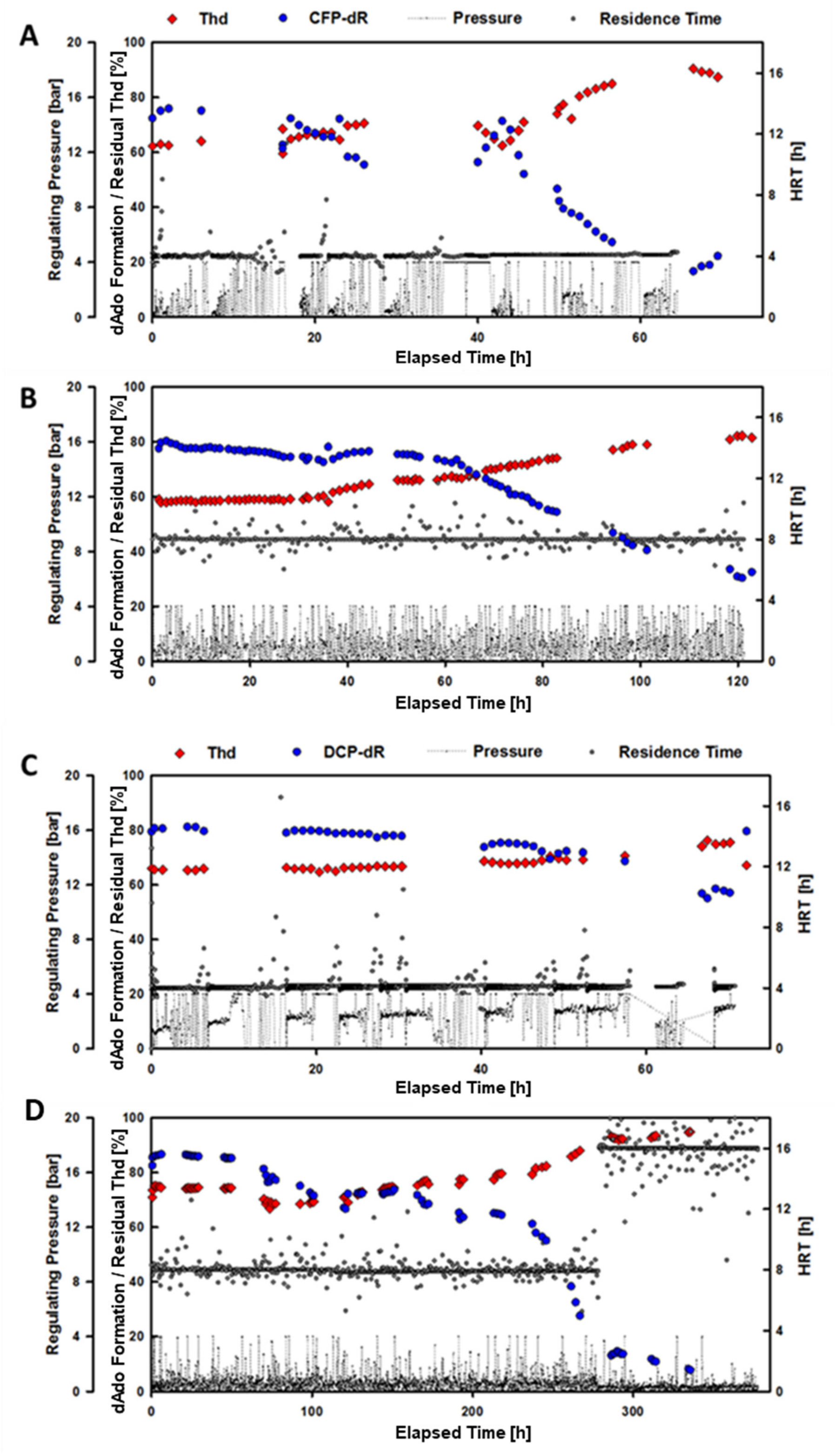

Figure S4. Transglycosylation reaction to produce the nucleoside analogues 6C2FP-dR **(A/B)** and 2,6DCP-dR **(C/D)** using purified NPs PyNP-02 and PNP-02. 25 mM of Thd and 10 mM of sugar acceptor were used as substrates and a reaction temperature of 40°C was applied. Sugar donor cleavage and nucleoside formation were observed in the EMR using a stirring speed of 200 rpm and a residence of 4 h (**A/C)** or 8 h (**B/D)** (and 16 h, **D**).

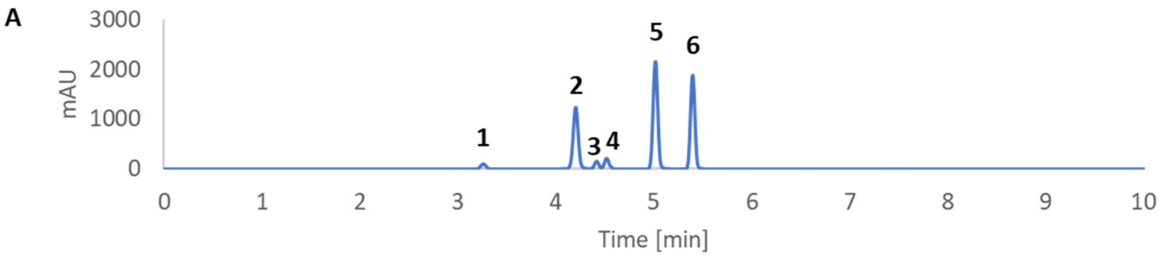

Peak	1	2	3	4	5	6
**Compound**	**Hypoxanthine**	**Thymine**	**dIno**	**Adenine**	**Thd**	**dAdo**
Peak area	424.2	4661.2	522.4	757.6	7297.3	6020.2
Conversion factor	4736.4	4089.9	435.8	6958.3	4830.1	7766.5
Dil. concentration [mM]	0.1	1.1	0.1	0.1	1.5	0.8
Concentration [mM]	0.9	11.4	1.2	1.1	15.1	7.8
Conversion Ade/ dAdo [%]				13		87
Conversion Thy/ Thd [%]		43			57	

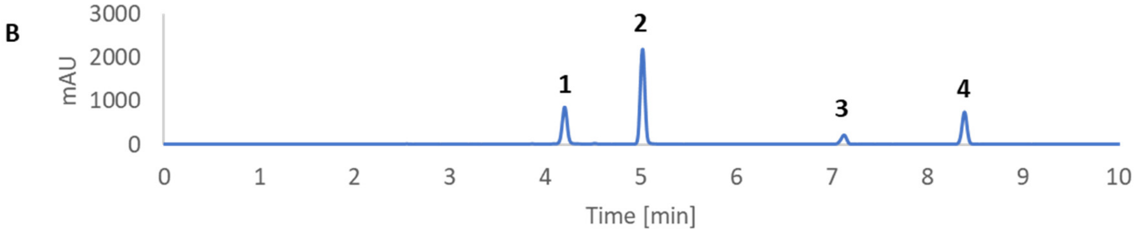

Peak	1	2	3	4
**Compound**	**Thymine**	**Thd**	**CFP**	**CFP-dR**
Peak area	3280.5	7549.3	901.2	2775.2
Conversion factor	4089.9	4830.1	3676.4	4037.5
Dil. concentration [mM]	0.8	1.6	0.3	0.7
Concentration [mM]	8.0	15.6	2.5	6.9
Conversion CFP/ CFP-dR [%]			26.3	73.8
Conversion Thy/ Thd [%]	33.9	66.1		

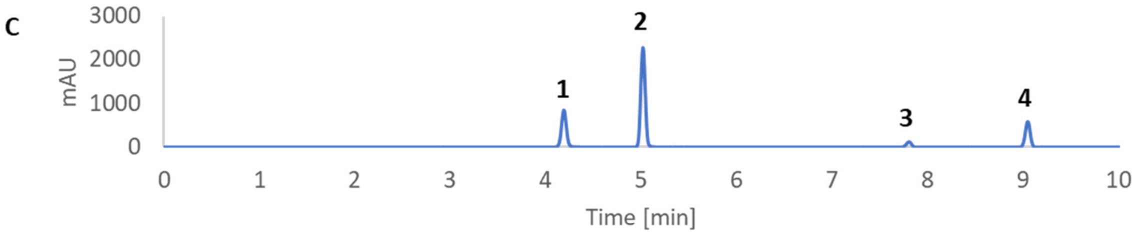

Peak	1	2	3	4
**Compound**	**Thymine**	**Thd**	**2,6DCP**	**26DCP-dR**
Peak area	3185.3	7568.2	494.9	2158.0
Conversion factor	4089.9	4830.1	2386	3164.7
Dil. concentration [mM]	0.8	1.6	0.2	0.7
Concentration [mM]	7.8	15.7	2.1	6.8
Conversion 2,6DCP/ 2,6DCP-dR [%]			23.3	76.7
Conversion Thy/ Thd [%]	33.2	66.8		

Figure S5. Calculation of the percentage conversion using example chromatograms for dAdo (A), 6C2FP-dR (B) and 2,6DCP-dR synthesis (C). The peak areas were converted to concentrations (dil. concentration) using conversion factors. By calculating the dilution factor (from the preparation of samples for HPLC) out, the concentration in the reaction was determined. The conversion [%] was then determined using equation 6.
